# Myosin XVI in the Nervous System

**DOI:** 10.3390/cells9081903

**Published:** 2020-08-15

**Authors:** Elek Telek, András Kengyel, Beáta Bugyi

**Affiliations:** 1 Department of Biophysics, Medical School, University of Pécs, Szigeti str. 12, H-7624 Pécs, Hungary; elek.telek@aok.pte.hu (E.T.); andras.kengyel@aok.pte.hu (A.K.); 2 Szentágothai Research Centre, Ifjúság str. 34, H-7624 Pécs, Hungary

**Keywords:** neural, development, myosin, mammalian, unconventional, myosin XVI, neurodegenerative

## Abstract

The myosin family is a large inventory of actin-associated motor proteins that participate in a diverse array of cellular functions. Several myosin classes are expressed in neural cells and play important roles in neural functioning. A recently discovered member of the myosin superfamily, the vertebrate-specific myosin XVI (Myo16) class is expressed predominantly in neural tissues and appears to be involved in the development and proper functioning of the nervous system. Accordingly, the alterations of *MYO16* has been linked to neurological disorders. Although the role of Myo16 as a generic actin-associated motor is still enigmatic, the N-, and C-terminal extensions that flank the motor domain seem to confer unique structural features and versatile interactions to the protein. Recent biochemical and physiological examinations portray Myo16 as a signal transduction element that integrates cell signaling pathways to actin cytoskeleton reorganization. This review discusses the current knowledge of the structure-function relation of Myo16. In light of its prevalent localization, the emphasis is laid on the neural aspects.

## 1. Introduction

Myosins are actin-based mechanochemical machines that convert the chemical energy of ATP hydrolysis to mechanical work and molecular movement along actin filaments [1,2]. Myosins are abundant in different types of tissues and involved in a large variety of cellular functions. Amongst these, several classes of the myosin superfamily (class I, II, III, V, VI, VII, IX, X, XV and XVI) are expressed in the nervous system [3,4]. Unequivocally, myosins play essential roles in the development and functioning of neural cells; and are implicated in diverse processes, such as neuroblast differentiation, neuronal migration, growth cone motility, axonal growth and transport or synaptic functions [3,4]. Myosins (myosin I, II, V, III, VI, VII, IX and XV) are important in the functioning, maintenance and morphology of sensory cells, including the hair cells in the inner ear, as well as the photoreceptors in the retina [3,4,5]. Based on the multiple functions of myosins in the nervous system, one can infer that they are implicated in human neurological disorders (Table 1). For a comprehensive overview of the neural functions of myosins, we direct the Readers to excellent reviews [3,4].

### Myosin XVI

**Figure 1:**
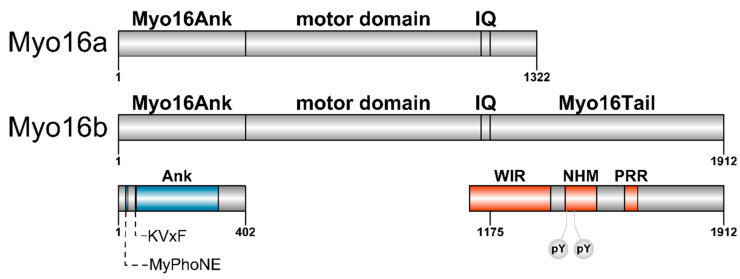
Domain structure of unconventional Myo16 isoforms. The number of amino acids corresponds to the rat Myo16 protein (accession number: Q9ERC1). Myo16a (1322 aa, 148.75 kDa) is composed of an N-terminal ankyrin domain (Myo16Ank) possessing a conserved myosin phosphatase N-terminal element (MyPhoNE), a KVxF sequence motif (protein phosphatase type 1 catalytic subunit (PP1c) binding motif) and eight ankyrin repeats (Ank). Myo16Ank is followed by the motor domain, a single IQ motif and a short C terminal region. The domain organization of the N-terminus of Myo16b (1912 aa, 210.56 kDa) is identical to that of Myo16a. Myo16b, following the IQ motif, has a longer C-terminal tail extension (Myo16Tail) including a WAVE1 interacting region (WIR), a neuronal tyrosine-phosphorylated adaptor for phosphoinositide 3-kinase (NYAP) homology motif (NHM) and proline-rich regions (PRR). The phosphotyrosines (Y^1416^ and Y^1441^) in the NHM motif are indicated (pY).

Myosin XVI (Myo16) as an unconventional myosin was discovered in a search for molecular motors associated to the motile events of nerve cells [26]. Myo16 is limited in distribution to the vertebrate lineage [27,28], and evolutionary related to the myosin III class [29]. The conserved motor domain of Myo16 is flanked by a unique combination of an N-terminal pre-motor extension (ankyrin domain, Myo16Ank) and a C-terminal tail (Figure 1). *MYO16* (cytogenetic location: 13q33.3) encodes two distinct protein isoforms generated by alternative splicing that differ in the length of their C-terminus; the shorter Myo16a and the longer, predominant Myo16b [26].

The expression and localization of Myo16 is prevalent in the central nervous system [26,30]. The expression of the principal Myo16b isoform in rats and mouse was detected from early embryonic stages throughout the lifespan of the animals. Its expression exhibits a developmental stage dependent pattern peaking in the first and second postnatal weeks [26]. The predominant expression of Myo16 in neural tissues suggests its importance in the proper functioning of the brain, mainly during development but also in adulthood. In line with this, the alterations of the human *MYO16*; either single nuclear polymorphisms (SNPs), deletions or epigenetic modifications are associated with neurodegenerative and neuropsychiatric disorders, including schizophrenia, autism spectrum disorder (ASD), bipolar disorder subtype II (BP-II) and major depressive disorder (MDD) (Table 1) [21,22,23,24,25]. In addition to the association of the *MYO16* gene to neurological disorders, many binding partners of Myo16 identified so far are central to normal brain functioning [31]. This implies that Myo16 may contribute to the functioning of the nervous system through its interactions with partner molecules.

Based on the recent biochemical and physiological analyses, Myo16 is emerging as an important regulator of the proper functioning of neural cells, although the exact molecular mechanisms underlying its role in the nervous system remain to be elucidated. Here, we provide an overview of the current understanding of the structural features, molecular interactions and cellular roles of Myo16 in the nervous system focusing on its unique domains: the N-terminal Myo16Ank and the C-terminal Myo16Tail. A more general overview of Myo16 can be found in *Myosins. A Superfamily of Molecular Motors* [31].

## 2. Interactions and Functions of Myosin XVI in the Nervous System

### 2.1. The N-terminal Myo16Ank Interacts with PP1c and Regulates Its Phosphatase Activity

Most of the myosins start with the conserved motor domain at the N-terminus of the polypeptide chain, but several classes (III, IX, XII, XV, XVI, XVIII) possess a pre-motor extension [29]. There are no structural or functional homologies among these pre-motor domains indicating that each evolved for a specific function [32,33,34,35,36]. The N-terminal extension of Myo16 (Myo16Ank) includes a myosin phosphatase N-terminal element (MyPhoNE), a KVxF motif and ankyrin repeats composed of tandem ankyrin motifs (Figure 1). These regions are conserved from fish to humans [31].

Based on its composition, Myo16Ank resembles the protein phosphatase 1 catalytic subunit (PP1c) binding site of the myosin phosphatase target/regulatory subunit 1 (MYPT1) of myosin light chain phosphatase (MLCP) [35]. MYPT1 is known to interact with PP1c [37]. The MYPT1:PP1c interaction is mediated through the KVxF motif, while the flanking regions provide additional contacts and tune the activity of the phosphatase [38]. Indeed, the association of Myo16 to the catalytic subunits PP1cα and PP1cγ was detected experimentally in postnatal rat cerebellum extracts in vivo, as well as by using recombinant proteins in vitro in surface plasmon resonance (SPR) studies **[26,35]**. Upon binding MYPT1 increases the dephosphorylation activity of PP1c towards the myosin regulatory light chain (RLC) target **[37]**. In contrast, the association of Myo16Ank to PP1c results in an opposite functional outcome; the phosphatase activity of the catalytic subunit towards the myosin RLC is significantly decreased in the Myo16Ank:PP1c complex in vitro [35]. Considering the large variability of the holoenzymes (see below) the opposite effect is not surprising.

The physiological relevance of this interaction and activity of Myo16Ank awaits further investigations, also targets of the Myo16Ank:PP1c have not been identified yet. PP1 is one of the most abundant protein phosphatases in eukaryotes; its catalytic subunit PP1c can interact with a large set of regulatory subunits in a mutually exclusive manner [38]. This results in the diversification of the holoenzymes with different specificity and functionality. Alternatively, the combinatorial control of the activity and substrate specificity of PP1c can be considered, whereby PP1c activity is increased or decreased depending on the combination of the regulatory subunits [39]. As an example, MYPT1 increases the activity of PP1c towards myosin RLC, but on the contrary, it decreases the PP1c activity towards glycogen phosphorylase [39]. The phosphorylation state of the target subunit can also affect the interaction and the functionality of the holoenzyme; serine and threonine phosphorylation of MYPT1 increases and decreases the phosphatase activity of the MYPT1:PP1c complex, respectively [38].

### 2.2. The-C terminal Myo16Tail Controls the Remodeling of the Actin Cytoskeleton by Linking PI3K and WAVE1

The C-terminal tails are highly versatile extensions among myosin classes containing different functional domains leading to diversified intracellular functions, such as anchoring, autoregulation, dimerization, cargo binding, cellular localization or protein-protein interaction [33,40,41,42]. In Myo16a isoform only a relatively short tail domain can be found at the C terminus, while a 590 amino acid tail extension (Myo16Tail, amino acids for rat Myo16) is a distinctive feature of the Myo16b isoform (Figure 1). The upstream region of Myo16Tail consists of a WAVE1 interacting region (WIR) followed by a neuronal tyrosine-phosphorylated adaptor for phosphoinositide 3-kinase (NYAP) homology motif (NHM) with two phosphorylation sites (Tyr^1416^ and Tyr^1441^) [30]. The downstream sequence of Myo16Tail possesses a considerable amount of structure-breaker proline residues and disorder-promoting amino acids [26,30,31,43]. These regions may attribute particularly important functions for Myo16Tail in the nervous system.

Myo16 was recently identified as NYAP3 amongst the neuronal tyrosine-phosphorylated adaptor for phosphoinositide 3-kinase proteins (NYAP 1-3) found in a screen for Src non-receptor tyrosine kinase family substrates by using a human hippocampus cDNA library [30]. The defining feature of the NYAP phosphoproteins is the presence of the NYAP homology motif (NHM) containing conserved tyrosine residues (YxxM) and a proline-rich stretch. Sequence analysis reveals the characteristic NHM motif in the C-terminal tail region of Myo16. It is specific for the Myo16b splice variant, the shorter Myo16a lacks this motif (Figure 1). In contrast to Myo16, NYAP1 and 2 do not possess any other regions N-terminally to their NHM [30].

**Figure 2:**
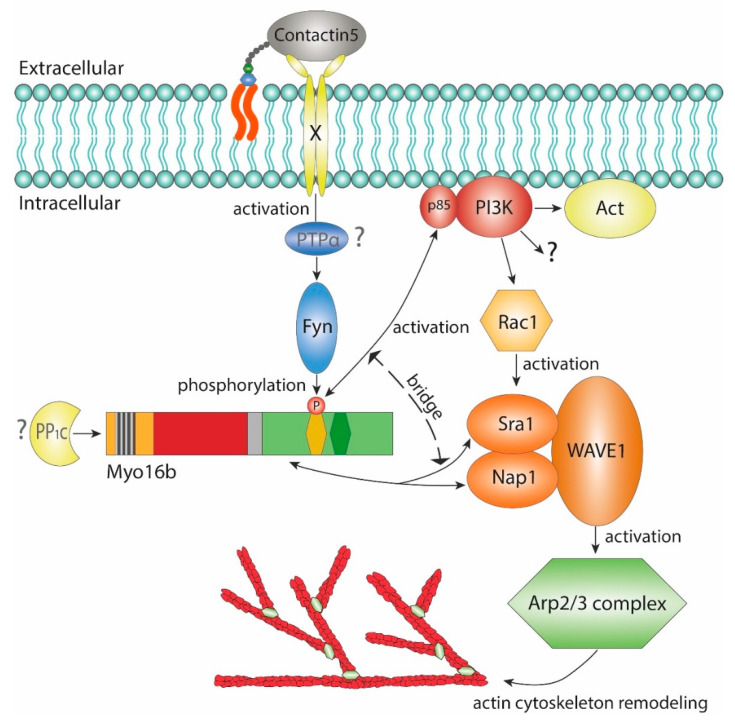
The C-terminal Myo16Tail in the reorganization of the actin cytoskeleton through the PI3K-WAVE1 pathway. Myo16Tail phosphorylation is triggered by Contactin5 through an unknown neuronal receptor (indicated with an X mark). Fyn binding to Myo16 is indirectly regulated by Contactin5 through a phosphatase (presumably via protein tyrosine phosphatase α (PTPα)). Tyrosine-phosphorylated Myo16 interacts directly with the p85 subunit of the phosphoinositide 3-kinase (PI3K) that is mediated by the NHM motif. The binding eventuates the activation of PI3K and its downstream effectors Akt and Rac1. Myo16 also interacts with the Sra1 and Nap1 subunits of WAVE1 through its WAVE1 interacting region. By simultaneous binding, Myo16 can bridge PI3K to WAVE1 and can contribute to the activation of WAVE1, thereby triggering the Arp2/3 complex-mediated remodeling of the actin cytoskeleton.

Myo16/NYAP3 was shown to be involved in signal transduction by linking the PI3K pathway to the remodeling of the actin cytoskeleton [30] (Figure 2). Myo16 is phosphorylated at the conserved tyrosine residues (Y^1416^xxM and Y^1441^xxM) in the NHM by the Src tyrosine kinase Fyn in the mouse brain [30]. Fyn is involved in transmitting signals from diverse transmembrane receptors and implicated in synaptic efficacy and plasticity (long-term potentiation), learning and memory [44]. Myo16 phosphorylation is stimulated by Contactin5, a glycosylphosphatidylinositol (GPI) anchored membrane protein. Contactins, a subfamily of the immunoglobulin superfamily of neural cell-adhesion molecules (Ig-CAMs) are important for neuritogenesis and synapse formation [45]. Contactins can activate Src tyrosine kinase Fyn indirectly via protein tyrosine phosphatase α (PTPα) suggesting a potential pathway of Fyn-induced Myo16 phosphorylation [46,47]. Tyrosine-phosphorylated Myo16 interacts with the p85 regulatory subunit of the phosphoinositide 3-kinase (PI3K). Of note, this mode of activation is reminiscent of the direct recruitment and activation of PI3K by receptor tyrosine kinases [48,49]. The formation of the Myo16:PI3K complex eventuates the activation of the p110 catalytic subunit of the kinase and that of its downstream effectors, Akt and Rac1 [30]. The Rho family GTPase Rac1 is a canonical activator of the WAVE regulatory complex (WRC) that activates the Arp2/3 complex, which, in turn, catalyzes the assembly of branched actin meshwork [50,51]. Interestingly, Myo16 also associates with the Sra1 and Nap1 components of the WAVE1 component of the regulatory complex through the WAVE1 interacting region (WIR) intercalated between the motor domain and the NHM **[30]**. In the ternary complex, Myo16 can link and potentiate PI3K-Rac1 to its downstream WRC, thereby it can contribute to the activation of the WRC-Arp2/3 complex and consequently to the regulation of the actin cytoskeleton. Indeed, transient transfection of HeLa cells with Myo16Tail resulted in the collapse of actin structures that required both the functional WIR and NHM [30].

**Figure 3:**
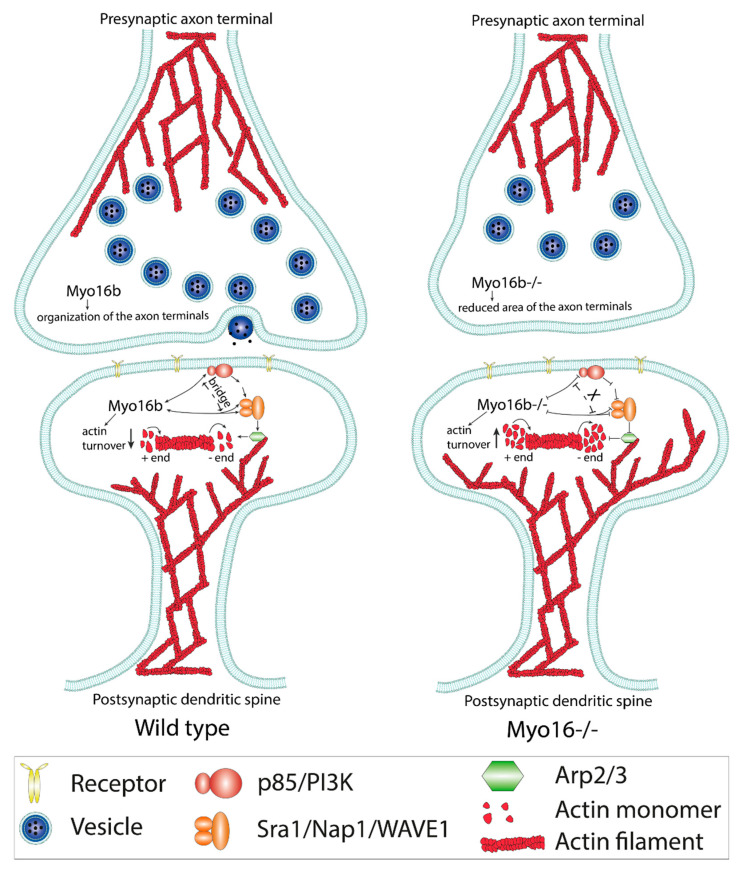
Schematic representation of the effects of Myo16 at parallel fiber-Purkinje cell synapses. Myo16 plays a role in the organization of the postsynaptic terminals of Purkinje cells by influencing the PI3K-WRC-Arp2/3 pathway that eventuates the downregulation of actin cytoskeleton dynamics. At the presynaptic site, Myo16 is implicated in the regulation of the number of synaptic vesicles, as well as of the morphological features of the parallel fiber terminal of granule cells. The X mark in the upper panel indicates the lack of interaction between Myo16, PI3K and WAVE1.

The physiological importance of the control of actin dynamics by Myo16 is further supported by studies in *MYO16^−/−^* mice [52]. Fluorescence recovery after photobleaching (FRAP) analysis of postsynaptic dendritic spines of Purkinje cells demonstrated that *MYO16* depletion leads to a faster F-actin turnover rate. A similar effect was observed upon inhibiting either the WRC or the Arp2/3 complex both in wild-type and *MYO16^−/−^* mice implying that Myo16 controls actin dynamics through this pathway. Altogether, both in vitro and in vivo data indicate that Myo16 by activating the WRC-Arp2/3 complex machinery functions to attenuate actin cytoskeleton dynamics. The data are consistent with a hypothesis in which Myo16 integrates signaling events to actin network reorganization, thereby it acts as an indirect regulator of the actin cytoskeleton in the dendritic spines of Purkinje cells. On the other hand, the phenotypic analysis of the cerebellar molecular layer of *MYO16^−/−^* mice revealed a significant decrease in the number of synaptic vesicles and the area of presynaptic terminals in parallel fibers of granule cells [52]. Comprehensively, Myo16 appears to be important for the regulation of actin dynamics at postsynaptic sites, as well as for the organization of presynaptic terminals; and therefore may be important in the normal structure, function and plasticity of granule cell-Purkinje cell synapses (Figure 3).

The physiological relevance of Myo16 in neural functioning was assayed in animal studies [30,52,53]. In the triple KO mice for all the three NYAP proteins including Myo16/NYAP3 a reduction in brain size and weight was observed that may be indicative of neurite hypotrophy [30]. In single knockout *MYO16* mice neither apparent abnormalities in the cerebellar anatomy nor change in selected synaptic markers were detected [52]. Behavioral analyses of *MYO16^−/−^* mice revealed no phenotypes and dysfunctions in locomotor activity, motor learning and social interaction; behaviors altered in the mouse model of ASD. Although the epigenetic control of *MYO16* was described upon experience-based learning (contextual fear conditioning) in mouse [53].

The NYAP1-3 proteins are encoded by different genes (chromosomal location: NYAP1 7q22.1, NAPY2 2q36.3, NYAP3 13q33.3) [54]. The three NYAP proteins share the NHM region characterized by the conserved tyrosine residues (YxxM) and a proline-rich stretch. They are all phosphorylated, and connect the PI3K activation to WAVE1 signaling and to the remodeling of the actin cytoskeleton [30]. Importantly, in contrast to NYAP1 and 2, Myo16/NYAP3 possesses a motor domain and ankyrin repeats N-terminally to the NHM. The combination of the NHM with other domains is expected to endow Myo16 with distinct functional characteristics. However, the specificity or redundancy of the activities of NYAPs has not been investigated with this respect. NYAP1 was isolated first in a screen performed with a GST-Lyn fusion protein [30]. The Src protein kinase Lyn is closely related to Fyn and can be activated directly by Contactin [46]. The Lyn-mediated pathway might have relevance in the regulation and diversification of the functioning of NYAPs.

## 3. Regulatory Mechanisms Controlling the Motor Function

In support of being a generic motor protein, Myo16b interacts with F-actin in an ATP-dependent fashion in rat brain (P8) extracts [26]. The F-actin association is further supported by the localization of GFP-Myo16 heavy chain to F-actin rich structures in the dendritic spines of Purkinje cells [52]. However, the kinetic features of the motor activity and the nature of the regulatory mechanism of the mechanochemical cycle of Myo16 has not been identified yet.

The mechanics of myosin motors can be tuned by many different mechanisms that provides myosin-specific regulation; including phosphorylation of the motor domain, interaction between myosin heavy and light chains, as well as ‘*backfolding*’ [55,56,57].

**Figure 4:**
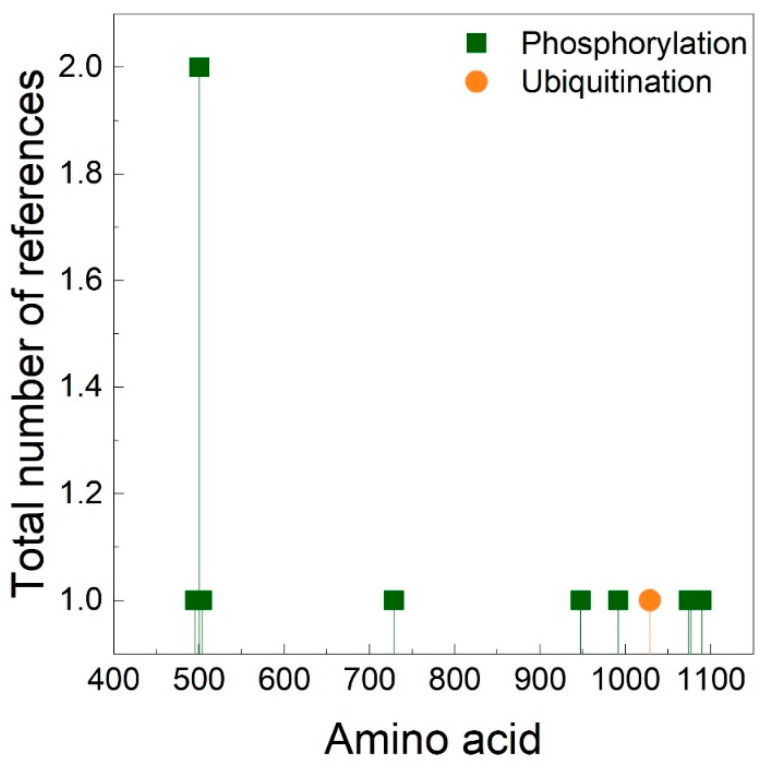
Post-translational prediction of Myo16 motor domain. Sequence analysis was performed using PhosphoSitePlus [71]. The total number of references shows the reliability of the prediction. Green squares indicate the phosphorylation sites while orange circle shows the ubiquitination site.

Phosphorylation of the heavy chain of the motor domain can control the enzymatic and mechanochemical activities of myosins. The consensus phosphorylation site designated as the TEDS site [56,57,58]. The head phosphorylation activates the ATPase activity of myosin I of lower eukaryotes in *Acanthaomeba* [59,60], *Dictyostelium* [61,62] and yeast [63]. By contrast, the ATPase activity of *Acanthamoeba* myosin II is down-regulated by phosphorylation of the motor domain at the motor-actin interface [64]. The motor domain of human myosin IIIA has a glutamic acid at the TEDS site (^660^Ex_16_DAMAK^680^). Although it is autophosphorylated by its kinase domain at two threonines in the loop 2 region (T^908^ and T^919^) [65,66,67]. The covalent modification considerably increases the ATPase activity, influences the steps of the ATPase cycle and negatively regulates the actin binding affinity of myosin IIIA. In mouse myosin VI, threonine in the TEDS site (^406^Tx_16_DALAK^426^) is phosphorylated, although it does not influence the ATPase activity of the motor domain [68]. Myo16 adheres to the TEDS rule (^722^Dx_16_DLLAK^742^) [26,31]. Although conserved serine and threonine phosphorylation sites can be predicted in the P-loop as the part of the ATP-binding pocket, preceding the DLLAK motif and in the C-terminus of the motor (Figure 4, Table 2) speculating that the motor domain of Myo16 is still object to regulation by phosphorylation, similarly to myosin IIIA.

Besides the predicted phosphoresidues a putative ubiquitination site is found at Lys^1029^. Ubiquitination triggers proteolysis and protein degradation through the ubiquitin-proteasome pathway in which they are recognized by the 26S proteasome and degraded [69]. In line with this, the 26S proteasome was implicated in the degradation of Myo16 [70].

The association between the IQ motif(s) of myosin heavy chain in the neck domain and the members of the calmodulin family; calmodulin or calmodulin-like (essential and regulatory) light chains can regulate both the structural properties and the mechanical adaptation of the myosin holoenzyme [55,73]. In this context, Myo16 has a single IQ motif in the neck region (Figure 1), however, the light chain of Myo16 has not been identified yet.

The ‘*backfolding*’ mechanism described in class IIa, V, VI, VII and X myosins provides an inhibitory regulation manifested through intramolecular interactions between the N-, and C-terminus of myosin adjoining the motor domain [74,75,76,77,78]. To investigate the potential intramolecular interactions and hierarchy of Myo16, the motor domain was modeled by using rabbit skeletal heavy meromyosin II (skHMM) or non-muscle myosin 2B [35]. Cosedimentation and SPR analysis showed that Myo16Ank binds skHMM with micromolar affinity and enhance the actin-activated Mg^2+^-ATPase of the motor domain [35]. Besides, experiments suggest that recombinant Myo16Ank and Myo16Tail can interact with a micromolar affinity (unpublished data) [43]. The above ranges of affinities are coincident with cellular functions, in which reversible interactions can occur having low affinity and high specificity [79]. In light of these molecular interactions, one can hypothesize that the motor domain of Myo16 may be upregulated directly by Myo16Ank, while the association of Myo16Tail and Myo16Ank may downregulate the motor activity indirectly, thereby providing control of the motor function in the cellular context.

## 4. The Importance of Polyproline Sequences and Phosphorylation Sites

Polyproline (PP) proteins comprise proline-rich motifs that tend to form polyproline helix II (PP II helix) [80]. PP II helices represent solvent-exposed and amphipathic binding interfaces that are frequently found in globular proteins [81,82]. Their occurrence is probably more common in intrinsically disordered proteins or protein regions (IDPs, IDRs) lacking a well-defined structural organization [80,83]. The proline-rich sequences by providing exposed and dynamic binding sites can serve for rapid recruitment and interchange of proteins, while tandem proline-rich motifs as part of adaptor systems can function in the assembly of protein interaction networks [80,84]. In support of this, several ligands prefer proline-containing regions, such as Src-homology 3 (SH3) and WW domain proteins, as well as Enabled (Ena)/vasodilator-stimulated phosphoprotein (VASP) homology 1 (Ena-EVH1), Homer-EVH1 and profilin [80]. On the other hand, phosphorylation sites are prevalent in proline-rich sequences and disordered protein regions **[85,86,87]**.

From this aspect, Myo16b possesses a considerable amount of structure-breaker proline residues, phosphosites and disorder-promoting amino acids in the C-terminal Myo16Tail [26,30,31]. In line with this, Myo16b is phosphorylated by Src family kinase Fyn at two phosphotyrosines (Y^1416^xxM and Y^1441^xxM) found in the NHM region of Myo16Tail [30]. The binding site of Fyn in Myo16 has not been identified yet. Src family of protein kinases possess consecutive SH2 and SH3 domains that mediate both intra-, and intermolecular interactions [88]. The SH3 interaction module consists of a peptide-binding pocket that can recognize ligands with a PxxP consensus motif, and also divergent, non-consensus target sites [89,90]. Myo16Tail contains canonical PxxP recognition sequences, the SH3 domain of Fyn might interact with these regions to position Myo16 adjacent to the SH1 tyrosine kinase domain. Phosphorylation of Myo16b eventuates the recruitment and activation of PI3K [30]. The SH2 domain of the p85 regulatory subunit binds preferentially phosphotyrosines in the Y^P^xxM phosphopeptide consensus sequence, indicating that the association of Myo16 and PI3K is likely to be mediated by this region [48]. In support of this, the interaction of Myo16-p85/PI3K was found to be dependent on the phosphorylation state of Myo16 [30].

Profilin is an actin-binding protein that also binds proline-rich ligands, such as formins, VASP and the WAVE family proteins [50,80,91,92]. The protein has four isoforms (PFN1-4), each is encoded by a distinct gene (*PFN1-4*). PFN1 is a ubiquitous isoform, while PFN2 shows predominant expression in the brain. Both PFN1 and 2 are important for actin-based neuronal processes; PFN1 regulates axonal growth and regeneration and PFN2 is associated with synaptic plasticity [93,94]. Since Myo16 is involved in the remodeling of the actin cytoskeleton and its C-terminus is rich in prolines, profilin appears to be an attractive candidate to associate with the Myo16-mediated processes. Profilin-binding proteins contain a recognition site of XP^5^ (X is G, L, I, S or A), either as a single copy or present in tandem repeats [92]. The very C-terminal region of Myo16Tail conforms to this consensus by possessing a P^5^ motif preceded by an alanine. Albeit, no direct interaction was found between recombinant Myo16Tail and PFN1 in steady-state anisotropy experiments [43]. This suggests the lack of or weak profilin binding, alternatively, the profilin isoform-specific nature of the interaction can be considered [95]. On the other hand, PFN1 is phosphorylated by RhoA-associated coiled-coil containing protein kinase 1 (ROCK1) at Ser^137^ in vivo that is conserved between the PFN1 and 2 isoforms [96]. ROCK phosphorylation as a regulatory switch influences the interactions of profilin with its ligands by increasing its affinity to polyproline sequences and actin [97]. Interestingly, the PP1cα catalytic subunit was identified to dephosphorylate PFN1 at Ser^137^ [98]. No direct association between PP1c and PFN1 was detected *in vitro*, suggesting that profilin is targeted by a PP1-interacting protein; however, regulatory subunits of PP1c for profilin modification have not been identified yet.

## 5. Concluding Remarks

The confined expression in neural tissues, the structural characteristics and interactions of the vertebrate-specific unconventional myosin XVI class suggest that it has an important role in neural development and functioning. As a support of this, the genetic examinations underscore the significance of Myo16 in the pathomechanism of neurological disorders, such as schizophrenia, autism spectrum disorder, bipolar disorder subtype II and major depressive disorder. The effects of the genetic alterations of *MYO16* on the function of the protein, as well as the molecular networks linking Myo16 to diseases, remain to be elucidated. Myo16 has a peculiar domain architecture that endows the protein with unique structural characteristics. As inherent for classic motor proteins, the direct actin interactions and the enzymatic characteristics of the isolated motor domain of Myo16 are still obscure. The generic motor domain of Myo16 is flanked by the N-terminal Myo16Ank and the C-terminal Myo16Tail extensions that appear to provide a molecular scaffold for versatile interactions. Myo16Ank interacts with the PP1c subunit and regulates its phosphatase activity [26,35]. This suggests that Myo16 may participate in the targeted dephosphorylation of proteins, however, the targets or the physiological relevance of this activity are not known yet. On the other hand, emerging evidence emphasizes that the C-terminus of Myo16 functions in linking the PI3K-WRC signaling pathway to actin cytoskeleton reorganization [30].

Altogether these findings indicate that Myo16 comprises a mechanism in which it acts as a signal transduction element. From this aspect, Myo16 resembles class III and IX myosins [32]. Significant recent advances started to decipher the molecular interactions and cellular functioning of Myo16, albeit, the exact physiological role of Myo16, the underlying molecular interactions and activities, as well as their spatio-temporal sequence, remain to be elucidated.

## Figures and Tables

**Table 1:** Association between different myosin classes and neurological disorders.

	Myosin ^1^						
	IIA IIB	IIIA	VA VB	VI	VIIA	XV	XVI
Myosin encoding gene	*NMMHC-IIA* *NMMHC-IIB*	*MYO3A*	*MYO5A* *MYO5B*	*MYO6*	*MYO7A*	*MYO15*	*MYO16*
Alzheimer disease	IIB						
Autism	IIB						XVI
Schizophrenia			VB				XVI
Bipolar disorder subtype II							XVI
Major depressive disorder							XVI
Non-syndromic deafness	IIA	IIIA			VIIA	XV	
Snell’s waltzer syndrome				VI			
Usher syndrome					VIIA		
Griscelli disease			VA				
Lissencephaly	IIB						
Sense of smell			VB				
*Reference*	[6,7,8]	[9]	[10,11,12]	[13]	[14,15,16,17,18]	[19,20]	[21,22,23,24,25]

^1^ A and B indicates the isoform of the myosin.

**Table 2:** Sequence alignment of Myo16 motor domain from different representatives of vertebrate classes in the aspect of post-translational modifications using Clustal-X [72].

Motor Domain							
Consensus Amino Acid ^1^	P-Loop	DLLAK Motif					
	(497–504 aa)	(722–742 aa)	S^948^	S^992^	K^1029^	Y^1075^GY^1077^	Y^1090^
	* * :	:	.	.	*	*	*
*Hs*	**S**[GERG**S**GK**S**]	DMIIRRH**T**IQIAEFFRDLLAK	**S**	**S**	**K**	**Y**G**Y**	**Y**
*Mm*	**S**[GERG**S**GK**T**]	DVIIRRH**T**IQMAAFYRDLLAK	**S**	**S**	**K**	**S**G**Y**	**Y**
*Rn*	**S**[GERG**S**GK**T**]	DVIIRRH**T**TQIAAFYRDLLAK	**S**	**S**	**K**	**Y**G**Y**	**Y**
*Gg*	**S**[GESG**S**GK**T**]	DMIVRRH**T**IEMAEFYRDLLAK	**S**	**S**	**K**	**Y**G**Y**	**Y**
*Xt*	**S**[GESG**S**GK**T**]	DMITRRH**S**VDTAEFYRDLLAK	**S**	**G**	**K**	**Y**G**Y**	**Y**
*Dr*	**S**[GESG**S**GK**S**]	DVITRRH**T**VEMSNHHRDLLTK	**C**	**N**	**K**	**Y**G**Y**	**Y**

Accession numbers of the sequences: *Homo sapiens* (*Hs*): Q9Y6X6, *Mus musculus* (*Mm*): Q5DU14, *Rattus norvegicus* (*Rn*): Q9ERC1, *Gallus gallus* (*Gg*): XP_416950.3, *Xenopus tropicalis* (*Xt*): A0A5G3IJG7, *Danio rerio* (*Dr*): F1QE80. Phosphorylation site prediction was analyzed in Figure 4. The conservation of the predicted phosphorylation/ubiquitination sites are labeled as fully conserved (*), strongly similar (:), weekly similar (.). Predicted phosphorylation sites and the ubiquitination site is colored by green and orange, respectively. ^1^Amino acid positions are given considering the sequence of the rat Myo16.
